# First-Line Treatment for Advanced Hepatocellular Carcinoma: A Three-Armed Real-World Comparison

**DOI:** 10.2147/JHC.S432948

**Published:** 2024-01-13

**Authors:** Robert Mahn, Oscar André Glüer, Farsaneh Sadeghlar, Christian Möhring, Taotao Zhou, Thomas Anhalt, Malte Benedikt Monin, Alexander Kania, Tim R Glowka, Georg Feldmann, Peter Brossart, Joerg C Kalff, Ingo G H Schmidt-Wolf, Christian P Strassburg, Maria A Gonzalez-Carmona

**Affiliations:** 1 Department of Internal Medicine I, University Hospital of Bonn, Bonn, Germany; 2 Department of Surgery, University Hospital of Bonn, Bonn, Germany; 3 Department of Internal Medicine III, University Hospital of Bonn, Bonn, Germany; 4 Department of Integrated Oncology CIO Bonn, University Hospital of Bonn, Bonn, Germany

**Keywords:** HCC, first-line therapy, subgroups

## Abstract

**Background and Aim:**

There are several existing systemic 1st- line therapies for advanced hepatocellular carcinoma (HCC), including atezolizumab/bevacizumab (Atez/Bev), sorafenib and lenvatinib. This study aims to compare the effectiveness of these three 1st-line systemic treatments in a real-world setting for HCC, focusing on specific patient subgroups analysis.

**Methods:**

A total of 177 patients with advanced HCC treated with Atez/Bev (n = 38), lenvatinib (n = 21) or sorafenib (n = 118) as 1st line systemic therapy were retrospectively analyzed and compared. Primary endpoints included objective response rate (ORR), progression-free survival (PFS) and 15-month overall survival (15-mo OS). Subgroups regarding liver function, etiology, previous therapy and toxicity were analyzed.

**Results:**

Atez/Bev demonstrated significantly longer median 15-month OS with 15.03 months compared to sorafenib with 9.43 months (p = 0.04) and lenvatinib with 8.93 months (p = 0.05). Similarly, it had highest ORR of 31.6% and longest median PFS with 7.97 months, independent of etiology. However, significantly superiority was observed only compared to sorafenib (ORR: 4.2% (p < 0.001); PFS: 4.57 months (p = 0.03)), but not comparing to lenvatinib (ORR: 28.6% (p = 0.87); PFS: 3.77 months (p = 0.10)). Atez/Bev also resulted in the longest PFS in patients with Child-Pugh A and ALBI 1 score and interestingly in those previously treated with SIRT. Contrary, sorafenib was non inferior in patients with impaired liver function.

**Conclusion:**

Atez/Bev achieved longest median PFS and 15-mo OS independent of etiology and particularly in patients with stable liver function or prior SIRT treatment. Regarding therapy response lenvatinib was non-inferior to Atez/Bev. Finally, sorafenib seemed to perform best for patients with deteriorated liver function.

## Introduction

Hepatocellular carcinoma (HCC) is the sixth most common cancer type worldwide and fourth leading cause of cancer death.^1^ The most important risk factor is liver cirrhosis. As clinical symptoms present late, it is mostly diagnosed at an advanced stage. Often a curative therapy is not possible anymore.

Palliative therapy of primary non resectable hepatocellular carcinoma follows according to the Barcelona classification (BCLC). In case of a macrovascular vessel-infiltration and/or extrahepatic metastases (BCLC C) without indication for local-interventional therapies or progressive disease after local therapies, a systemic therapy is the standard care. Sorafenib, a tyrosine-kinase inhibitor, first demonstrated a survival benefit in the 2007 published SHARP Study with an overall survival of 10.7 months, extending the progression-free survival to 5.5 months which was 2.7 months longer than placebo treatment.^2^ In 2018 lenvatinib, another tyrosine-kinase-inhibitor, showed a non-inferiority in overall survival compared to sorafenib in the REFLECT-Study (13.6 vs 12.3 months) and a significant prolongation of PFS (7.4 vs 3.7 months), leading to approval of lenvatinib for equal use as sorafenib in first-line palliative treatment.^3^ In 2020 the use of the PD-L1-inhibitor atezolizumab in combination with the VEGF-inhibitor bevacizumab was also approved as a new first-line therapy for advanced HCC. The Phase III IMbrave150-Study demonstrated significant increase of median overall survival, which was not reached in the observation period and progression-free survival (6.8 vs 4.3 months) compared to sorafenib.^4^ Most recently, a combined immune checkpoint inhibition with tremelimumab (Anti-CTLA-4) and durvalumab (Anti-PD-L1) was also approved as a further systemic first-line therapy after displaying superiority over sorafenib in the Phase III randomized HIMALAYA trial.^5^,^6^

Those prospective trials excluded patients with impaired liver function like Child-Pugh B and patients with reduced ECOG, which raises questions about efficacy of systemic therapy for these patients. While there is real-world data comparing Atez/Bev with sorafenib and lenvatinib separately, a comparative study of all three first-line therapies within a single cohort is missing.^7^

The development of immune checkpoint inhibitors for treatment of advanced HCC unfolds new opportunities and raises questions regarding real-world performance and which individual patient characteristics and pre-treatments influence the outcome.^8–11^

This retrospective single-center study aims to fill this gap, comparing these three palliative therapies in an unselected real-world cohort of patients with advanced HCC, also within selected subgroups, regarding liver function, etiology and previous therapy; to figure out who is doing well with which therapy.

## Patients & Methods

### Patients

Patients were selected out of a database of approximately 1000 patients with HCC treated at the Comprehensive Cancer Center (CIO Bonn) of the University Hospital of Bonn, Germany between 2004 and 2022. We considered for inclusion patients diagnosed with HCC who had no previous systemic therapies and were either ineligible for further surgery or locoregional therapies.

According to these criteria 177 patients were eligible for inclusion. Of these, 118 received systemic treatment with sorafenib, 21 with lenvatinib and 38 with atezolizumab plus bevacizumab (Atez/Bev).

### Study Design

This is a single-center, retrospective study analyzing a real-world and unselected cohort of patients with advanced HCC, who had not previously undergone systemic chemotherapy. Patients receiving either sorafenib, lenvatinib, or atezolizumab in combination with bevacizumab as first-line therapy were analyzed and compared. Initial analysis involved a global comparison of all groups, followed by separate comparative evaluations of the Atez/Bev group against both sorafenib and lenvatinib groups individually. Baseline parameters (refer to Table 1) were recorded prior therapy start. Patients who were lost to follow-up were censored at the date of their last known follow-up.

**Table 1:** Baseline Parameters

Variable	All (n=177)	Sorafenib (n=118)	Lenvatinib (n=21)	Atez/Bev (n=38)	Global p-value	p-value Sorafenib vs Atez/Bev	p-value Lenvatinib vs Atez/Bev
Age	66 (58–73)	66 (58–72)	67 (61–73)	68 (59–74)	0.679		
Male	136 (76.8%)	94 (79.7%)	12 (57.1%)	30 (78.9%)	0.075		
BMI	26 (23–30)	26 (23–29)	26 (24–30)	27 (24–30)	0.985		
Weight	81 (72–95)	80 (74–95)	75 (72–100)	84 (71–95)	0.641		
**ECOG**					0.510		
0/1	133 (75.1%)	82 (69.5%)	17 (80.9%)	34 (89.5%)			
≥2	44 (24.9%)	36 (30.5%)	4 (19.0%)	4 (10.5%)			
**Child-Pugh-Class**					0.028	0.025	1.000
A	109 (61.6%)	63 (53.4%)	16 (76.2%)	30 (78.9%)			
B/C	60 (33.9%)	47 (39.8%)	5 (23.8%)	8 (21.1%)			
Unknown	8 (4.5%)	8 (6.8%)	0	0			
**Etiology**							
Presence of Cirrhosis	150 (84.8%)	104 (88.1%)	18 (85.7%)	28 (73.7%)	0.099		
Alcohol	42 (23.7%)	29 (24.6%)	9 (42.9%)	4 (10.5%)	0.019	0.096	0.012
HBV	30 (17.0%)	19 (16.1%)	2 (9.5%)	9 (23.7%)	0.351		
HCV	47 (26.6%)	37 (31.4%)	3 (14.3%)	7 (18.4%)	0.118		
NASH	31 (17.5%)	11 (9.3%)	9 (42.9%)	11 (29.0%)	0.000	0.009	0.426
Other	38 (21.5%)	30 (25.4%)	3 (14.3%)	5 (13.2%)	0.194		
**BCLC**					0.000	0.002	<0.001
B	38 (21.5%)	21 (17.8%)	0	17 (44.7%)			
C	138 (78.0%)	96 (81.4%)	21 (100.0%)	21 (55.3%)			
D	1 (0.6%)	1 (0.8%)	0	0			
Macrovascular invasion of main vessel	51 (28.8%)	28 (23.7%)	10 (47.6%)	13 (34.2%)	0.359		
Extrahepatic Metastases	91 (51.4%)	62 (52.5%)	15 (71.4%)	14 (36.8%)	0.041	0.092	0.022
Maximal size of intrahepatic tumor. cm	6 (3–9)	6 (4–9)	6 (4–8)	5 (3–8)	0.629		
**Grading**					0.299		
1	14 (7.9%)	11 (9.3%)	0	3 (7.9%)			
2	67 (37.8%)	42 (35.6%)	10 (47.6%)	15 (39.5%)			
3	21 (11.9%)	12 (10.2%)	4 (19.0%)	5 (13.2%)			
MELD-Score	8 (7–11)	9 (7–12)	7 (6–8)	8 (8–10)	0.010	0.267	0.030
ALBI-Score					0.007	0.009	1.000
1	49 (27.7%)	24 (20.3%)	8 (38.1%)	17 (44.7%)			
2	84 (47.5%)	57 (48.3%)	11 (52.4%)	16 (42.1%)			
3	33 (18.6%)	27 (22.9%)	1 (4.8%)	5 (13.2%)			
**Crafity-Score**					0.027	0.017	0.027
0	69 (39.0%)	39 (33.1%)	7 (33.3%)	23 (60.5%)			
1	73 (41.2%)	49 (41.5%)	14 (66.7%)	10 (26.3%)			
2	5 (2.8%)	4 (3.4%)	0	1 (2.6%)			
AFP-values	84 (10–1980)	173 (12–2389)	140 (13–5061)	26 (6–402)	0.173		
AFP >400ng/mL	69 (40.8%)	50 (44.2%)	10 (47.6%)	9 (25.7%)	0.121		
**Previous therapies**							
Surgery	52 (29.4%)	32 (27.1%)	10 (47.6%)	10 (26.3%)	0.149		
RFA/MWA/HIFU	14 (7.9%)	5 (4.2%)	2 (9.5%)	7 (18.4%)	0.019	0.019	0.611
SIRT	39 (22.0%)	23 (19.5%)	1 (4.8%)	15 (39.5%)	0.005	0.026	0.006
TACE	68 (38.4%)	45 (38.1%)	11 (52.4%)	12 (31.6%)	0.291		
Transplantation	4 (2.3%)	3 (2.5%)	1 (4.8%)	0	0.471		
TIPS	13 (7.3%)	10 (8.5%)	1 (4.8%)	2 (5.3%)	0.717		
Treated varices at baseline	71 (40.1%)	52 (44.1%)	5 (23.8%)	14 (36.8%)	0.198		
**Second Line Therapy**							
Systemic Second-Line	48 (27.1%)	26 (22.0%)	12 (57.1%)	10 (26.3%)	<0.001	0.001	0.627

**Notes**: Numerical data are presented as median with range in parenthesis. Categorial data are presented as absolute frequency with relative frequency in parenthesis.

**Abbreviations**: ECOG, Eastern Cooperative Oncology Group; HBV, hepatitis B; HCV, hepatitis C; NASH, non-alcoholic steatohepatitis; BCLC, Barcelona Clinic Liver Cancer; RFA, Radiofrequency Ablation; MWA, microwave ablation; HIFU, high-intensity focused ultrasound; SIRT, selective internal radiation therapy; TACE, transarterial chemoembolization; TIPS: transjugular intrahepatic portosystemic shunt.

To ensure comparability among the groups, we restricted the timeframe for OS and PFS to 15 months post initiation of therapy, given the relatively recent introduction of atezolizumab plus bevacizumab in comparison to other drugs. Diagnosis was confirmed either through radiologic imaging, which included computed tomography or magnetic resonance imaging (70 patients), or via histological evaluation (107 patients). Tumor staging was determined based on radiologic imaging according to the Response Evaluation Criteria in Solid Tumors (RECIST) 1.1. Any adverse events that occurred during first-line therapy were documented, and severity was defined by CTCAE version 5.0. Ethical approval for this retrospective study and waiver of consent for participation in the study for all patients/using patient details was granted by the Ethics Committee of the Medical Faculty of the University of Bonn (No. 341/17). All research was conducted in accordance with both the Declarations of Helsinki and Istanbul. Written consent for therapy was given in writing by all subjects. If patient was alive, they gave written consent for this retrospective analysis.

### Therapy Decision

Therapeutic options were discussed in our interdisciplinary tumour conference at a high-volume liver cancer center. Generally, patients were considered inoperable because of advanced stage of their disease and/or associated with impaired liver function, severe comorbidities, or a low performance status. Patients who were not suitable candidates for surgery were initially considered for local therapies, such as transarterial chemoembolization (TACE), radiofrequency ablation (RFA), or selective internal radiation therapy (SIRT), according with the standard guidelines of the European Society of Medical Oncology (ESMO), the European Association for the Study of the Liver (EASL) and German standard guidelines for HCC.^12^,^13^ Before 2018, sorafenib was the only available systemic treatment. However, with the approval of additional therapeutic options, patients were treated according to the most suitable drug based on their comorbidities and liver function. These drugs included sorafenib, lenvatinib, or atezolizumab in combination with bevacizumab in the first line therapy, and additional cabozantinib, regorafenib or ramucirumab in further lines of therapy. Moreover, nivolumab or pembrolizumab were use as off-level uses in further lines of therapy when standard treatment options had been exhausted in further lines of therapy. Each therapeutic decision was made in agreement with the individual patient, taking into account their preferences and potential treatment toxicity.

### Statistical Analysis

Patient data were retrieved from the electronic in-house patient database (AGFA HealthCare ORBIS) and organized and pseudonymized in a database before statistical analysis.

The Kruskal–Wallis equality of populations rank test was used to compare the three subgroups. For comparisons involving only two groups, the two-sample exact Wilcoxon rank-sum test and the two-sample test of proportions were utilized. The Fisher’s exact test was used for the statistical analysis of response rates.

Numerical data are presented as median with range in parentheses, unless otherwise indicated. Categorial data are presented as absolute frequency with relative frequency in parenthesis, unless otherwise indicated. Hazard ratios were only calculated with simple Cox regression analysis using univariate time-to-event-analysis, as according to Babyak the n in the subgroups was too small for a resilient multivariate analysis.^14^ This approach was also used to identify independent predictors.

Survival was compared by Log rank test and transcribed into Kaplan–Meier diagrams. P-values are presenting the global comparison, unless otherwise specified. P-values ≤0.05 were considered statistically significant. Sorafenib and lenvatinib were compared with the atezolizumab/bevacizumab subgroup in the case of significant global tests in baseline and generally in survival comparisons. All statistical analyses were performed using STATA Statistics/Data analysis versions 16.1 and 17.1 (StataCorp LLC, College Station, TX, USA).

## Results

### Baseline Characteristics

Baseline characteristics are presented in Table 1. Analyzing all patients of our cohort, 150 patients (84.8%) had a cirrhosis, as an etiology we could identify alcohol in 42 patients (23.7%), hepatitis B in 30 patients (17%), hepatitis C in 47 patients (26.6%) and NASH in 31 patients (17.5%). A total of 109 patients (61.6%) had Child-Pugh-Class A, 108 patients (61.0%) had an ECOG status of 1 or 2. Further, 138 patients (78.0%) were classified as BCLC C, and 38 patients (21.5%) as BCLC B. The median maximal tumor size was 6 cm. Elevated AFP levels (≥400ng/mL) were observed in 69 patients (40.8%) at the beginning of 1st-line treatment. Histological analysis of 102 patients revealed 67 patients (37.8%) with moderately differentiated tumor grade 2 and 21 patients (11.9%) with poorly differentiated tumor grade 3. Among the patients, 52 patients (29.4%) previously underwent surgery, 14 patients (7.9%) received ablative therapies, such as RFA, MWA or HIFU, 39 (22%) patients were treated with SIRT, 68 (38.4%) patients had undergone TACE, and four patients (2.3%) had a transplantation as previous therapy. Of notice, some patients exhibited multiple etiologies or multiple prior therapies.

Comparing liver function based on Child-Pugh-Class among all three groups, the sorafenib group displayed statistically significant poorer liver function with 47 patients (39.8%) having a Child-Pugh-Class B/C, compared to the Atez/Bev group with 8 patients (21.1%) with Child-Pugh B/C (p = 0.03). By contrast, the median MELD-Score of the Atez/Bev patients (8) was statistically significantly higher than that of the lenvatinib group (7) (p = 0.03). Significant differences in the ALBI-Score were observed between the sorafenib and Atez/Bev groups, particularly when comparing ALBI Score = 1 (sorafenib group: 24 patients (20.3%); Atez/Bev group: 17 patients (44.7%)). Regarding tumor stage, a significant difference was noted in the BCLC stage between the Atez/Bev and lenvatinib groups (p = 0.02), with all patients in the lenvatinib group classified as BCLC C. Again, there was a statistically significant difference between the Atez/Bev and sorafenib groups in BCLC stage (p = 0.002). The prevalence of metastatic disease differed significantly among the Atez/Bev group (36.8%) and the lenvatinib group (71.4%), with less frequent metastasis observed in the Atez/Bev group (p = 0.02). A significantly higher number of patients in the Atez/Bev group had undergone previous local therapies such as RFA/MWA and SIRT compared to the lenvatinib group. No significant differences were observed between the three groups regarding other baseline parameters like AFP, and other previous therapies such as surgery or treated varices.

### Efficacy of Atezolizumab/Bevacizumab Compared to Sorafenib or Lenvatinib

Response rates are presented in Table 2. A statistically significant difference was observed when comparing the response across the three patient groups. The highest ORR was achieved in the Atez/Bev group with 31.6%, followed by the lenvatinib group with 28.6% and the sorafenib group with 4.2%. There was a statistically significant difference between Atez/Bev and sorafenib (p < 0.0001), but not between Atez/Bev and lenvatinib (p = 0.87). Of note, the only patient who achieved complete response in our trial was from the Atez/Bev group (2.6%). Rates for the disease control (DCR) are also best for Atez/Bev (55.3%), followed by lenvatinib (42.9%) and sorafenib (19.5%) with statistically significant difference for Atez/Bev against sorafenib (p < 0.0001) but not against lenvatinib (p = 0.78).

**Table 2:** Response Rates

Response	Sorafenib Group	Lenvatinib Group	Atez/Bev Group	Global p-value	p-value Sorafenib vs Atez/Bev	p-value Lenvatinib vs Atez/Bev
**Complete response**	0	0	1 (2.6%)	<0.001	0.077	0.453
**Partial response**	5 (4.2%)	6 (28.6%)	11 (28.9%)	<0.001	<0.001	0.976
**Stable disease**	18 (15.3%)	3 (14.3%)	9 (23.7%)	<0.001	0.232	0.391
**Progressive disease**	79 (66.9%)	9 (42.9%)	17 (44.7%)	<0.001	0.014	0.889
**Objective response rate**	5 (4.2%)	6 (28.6%)	12 (31.6%)	<0.001	<0.001	0.870
**Disease control rate**	23 (19.5%)	9 (42.9%)	21 (55.3%)	<0.001	<0.001	0.779

**Note**: Data are presented as absolute frequency with relative frequency in parenthesis.

**Figure 1:**
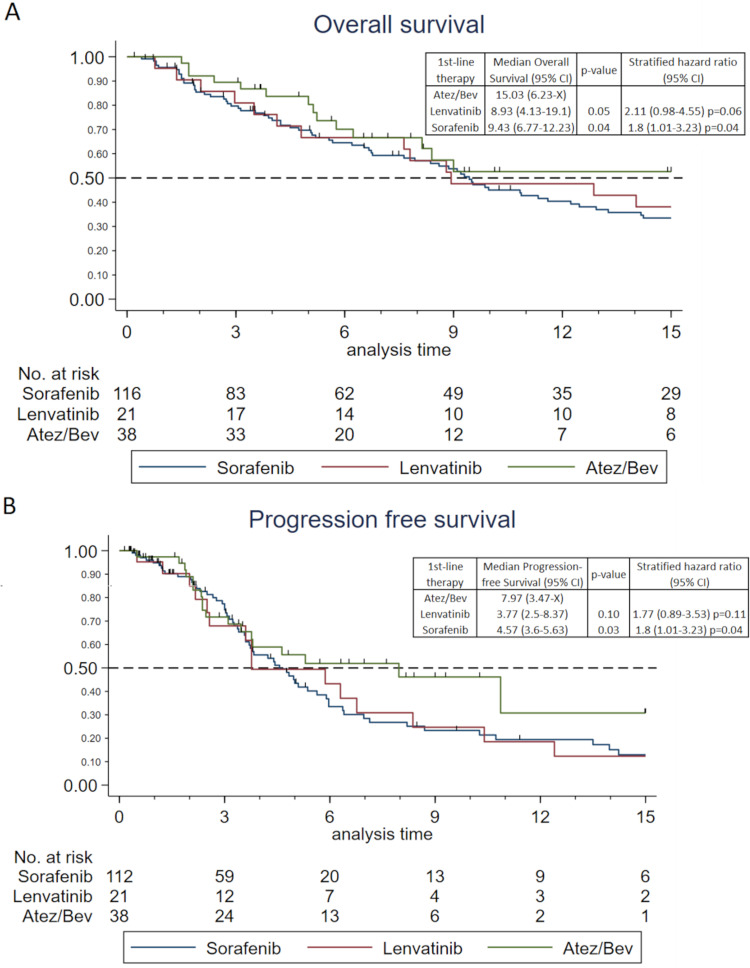
“Overall and progression free survival” (**A**) Kaplan-Meier graph of overall survival: p=0.088 (Log rank test) (**B**) Kaplan-Meier graph of progression free survival: p=0.075 (Log rank test). Global p-values are shown. Tick marks indicate censored data.

Overall survival (OS) and progression free survival (PFS) are depicted in Figure 1. No significant global difference was observed for the 15-month OS across all three therapies (p = 0.09). However, Atez/Bev demonstrated superior 15-month OS compared to the other groups. The longest median 15-month OS [15.03 months (95% CI: 6.23-x)] was achieved with Atez/Bev, presenting a statistically significant difference when compared with sorafenib [9.43 months (95% CI: 6.77–12.23), p=0.04] and lenvatinib [8.93 months (95% CI: 4.13–19.1), p = 0.05], the latter having the shortest median 15-month OS.

Global PFS was not statistically significant either (p = 0.08). However, Atez/Bev was also significantly superior to sorafenib (p = 0.03) but not to lenvatinib (p = 0.10). The Atez/Bev group also had the longest median PFS time at 7.97 months (95% CI: 3.47-x). The median PFS of the sorafenib group was 4.57 months (95% CI: 3.6–5.63), while the lenvatinib group reported the shortest median PFS at 3.77 months (95% CI: 2.5–8.37).

### Subgroup Analysis

**Figure 2:**
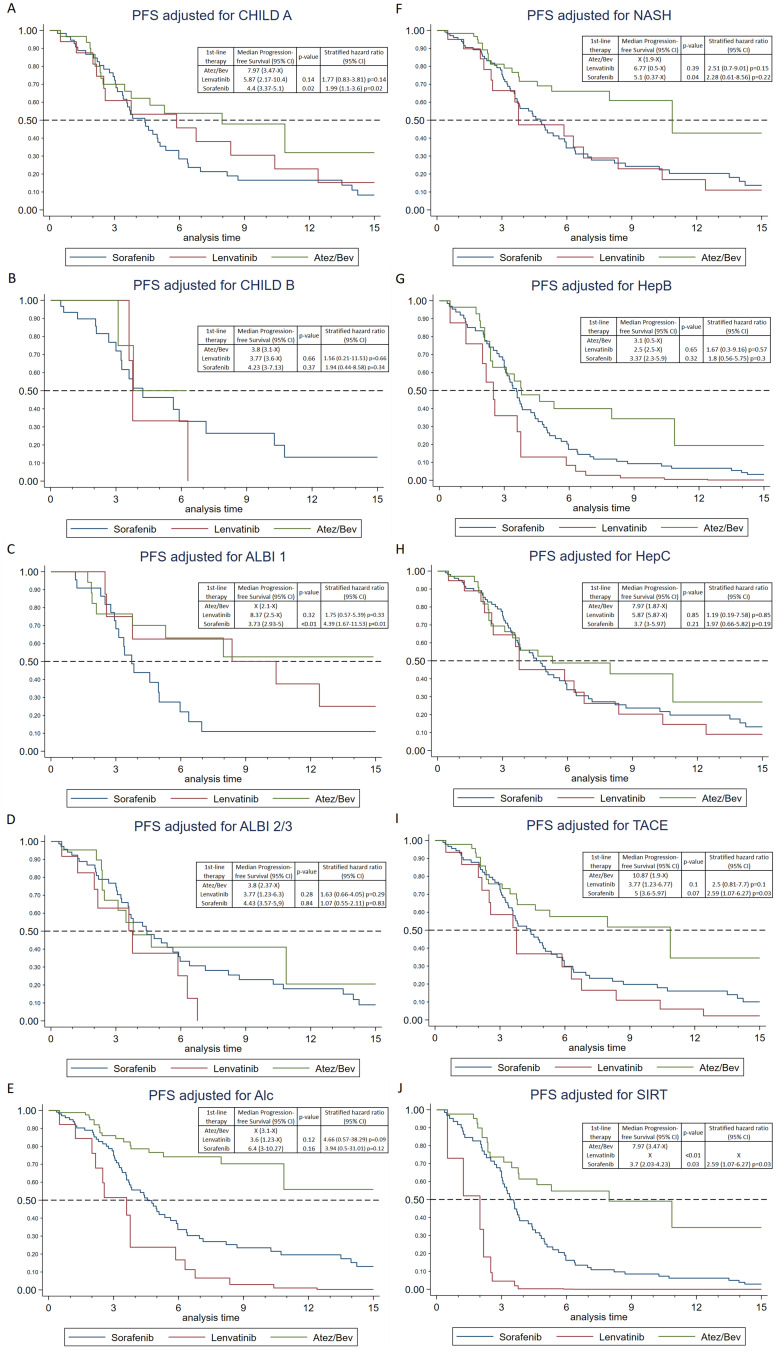
Progression free survival for different subgroups (**A**) CHILD A; (**B**) CHILD B; (**C**) ALBI 1; (**D**) ALBI 2/3; (**E**) Alc, Alcohol; (**F**) NASH, non-alcoholic steatohepatitis; (**G**) HepB, hepatitis B; (**H**) HepC, hepatitis C; (**I**) TACE, transarterial chemoembolization; (**J**) SIRT, selective internal radiation therapy; (A-J, each Log rank test, CI: confidence interval) p-values and hazard ratios are for direct comparison to Atez/Bev.

The results of the subgroup analysis for Progression-Free Survival (PFS) are presented in Figure 2. Overall, PFS was not statistically significant either for Child-Pugh Class A (p = 0.06) or for Child-Pugh Class B (p = 0.68) comparing all three groups of therapy. Upon individual comparison, the data reveal that Atez/Bev yields again a significantly longer median PFS, particularly with better liver function when compared against sorafenib for Child-Pugh Class A [7.97 months for Atez/Bev (95% CI: 3.47-x) versus 4.4 months for sorafenib (95% CI: 3.37–5.1), p = 0.02]. However, this comparison was not significant against lenvatinib [Child-Pugh A: 5.87 months (95% CI: 2.17–10.4), p = 0.14]. Individual comparisons for Child-Pugh B were also not statistically significant and all therapies performed worse than with Child-Pugh A. Yet, for Child-Pugh B, sorafenib showed a slightly longer median PFS with 4.23 months (95% CI: 3–7.13), compared to lenvatinib (3.77 months (95% CI: 3.6-x)) and Atez/Bev (3.8 months (95% CI: 3.1-x)).

Interestingly, regarding ALBI score global comparison was statistically significant (p = 0.004) for ALBI Grade 1, but not for ALBI Grades 2/3 (p = 0.49). Moreover, the individual comparison of patients with ALBI Grade 1 showed a statistically significant superiority of Atez/Bev over sorafenib in terms of PFS [PFS not reached within 15 months for 50% in Atez/Bev (95% CI: 2.1-x) versus sorafenib: 3.73 months (95% CI: 2.93-x), p = 0.001]. However, this superiority was not observed when compared to lenvatinib [ALBI 1: 4.69 months (95% CI: 2.5-x), p = 0.32]. Direct comparisons among patients presenting ALBI Grades 2/3 when starting first-line therapy were not statistically significant. Again, in this group, we observed a general decrease in PFS for all treatments. However, sorafenib showed a slight increase in PFS to 4.43 months (95% CI: 3.57–5.9) compared to lenvatinib with 3.77 months (95% CI: 1.23–6.3) and Atez/Bev 3.8 months (95% CI: 2.37-x).

We also examined the PFS across different etiologies. Global and individual comparison revealed no statistically significant difference for all etiologies among the groups, nevertheless the longest PFS was consistently observed in the Atez/Bev group, including the group of NASH patients.

Among all prior local therapies, only Selective Internal Radiation Therapy (SIRT) demonstrated a statistically significant difference between the groups (p = 0.004). In this subgroup, Atez/Bev had the longest PFS (7.97 months) compared to the other groups, which was statistically significant against sorafenib (3.7 months, p = 0.03) and lenvatinib (p < 0.001). Previous Transarterial Chemoembolization (TACE) also extended the median PFS to 10.87 months, albeit not statistically significant to the other groups.

### Univariate Analysis

**Figure 3:**
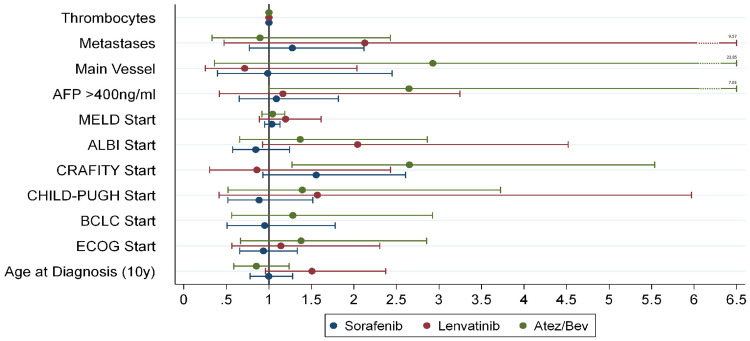
Hazard ratios of individual parameters regarding PFS prediction depicted with 95% confidence intervals after using the Cox-proportional hazards model. Values >1 indicate a bad influence on PFS with higher grade of the parameter. Exception are the thrombocytes with lower values increasing the hazard ratio. Age is grouped in 10-year steps for better representability. Main vessel means macroscopic main vessel infiltration. (Cox Regression).

**Abbreviations**: BCLC, Barcelona Clinic Liver Cancer; ECOG, Eastern Cooperative Oncology Group.

Univariate analysis of baseline characteristics was performed to identify significant predictors for progression free survival (PFS). Results are displayed in Figure 3.

The univariate time-to-event analysis showed a statistically significant correlation between PFS and therapy decision regarding Atez/Bev versus sorafenib (HR: 0.57; [95% CI: 0.34–0.97]; p = 0.04) during the observation period. However, no statistically significant difference was noted against lenvatinib, although Atez/Bev versus lenvatinib also demonstrated a reduced hazard ratio (HR: 0.56; [95% CI: 0.28–1.12]; p = 0.10).

Looking at selected baseline parameters without time limit, the only statistically significant identified predictor of PFS was the CRAFITY-Score, exclusively for Atez/Bev (HR: 2.65; 95% CI: 1.27–5.54; p = 0.01). The other analyzed parameters showed no statistically significant difference. Nonetheless, metastases exhibited the highest HR for lenvatinib, reaching with the 95% CI up to 9.57, and main vessel infiltration for Atez/Bev, increasing the 95% CI up to 23.85, along with higher Alpha-Fetoprotein (AFP) values, raising the 95% CI up to 7.03. Furthermore, in comparison, lenvatinib manifested the highest 95% CI values with decreasing liver function, as determined by Child-Pugh Class, MELD Score, and ALBI Grade.

### Feasibility and Toxicity

The distribution of adverse events is presented in Table 3. Treatment-related grade 3 or higher adverse events occurred in 63 patients (53.4%) of the sorafenib group, in 5 patients (23.8%) of the lenvatinib group and in 7 patients (18.4%) of the Atez/Bev group. Atezolizumab plus bevacizumab showed the lowest overall rate of adverse events but had the highest rate of immune related adverse events. Two patients (5.3%) experienced severe immune-related AEs; one developed an autoimmune hepatitis and the other experienced corneal transplant rejection. Additionally, Grade 3 or higher colitis was observed in two patients (5.3%). The most common severe AEs in the sorafenib group were fatigue with 22 patients (18.6%) and nausea/emesis with 21 patients (17.8%). In the lenvatinib group the most common severe AE was pulmonary embolism in two patients (9.5%).

**Table 3:** Frequency of Adverse Events

Adverse Events	Sorafenib		Lenvatinib		Atez/Bev	
	(N=118)		(N=21)		(N=38)	
	Grade ≥3	Any Grade	Grade ≥3	Any Grade	Grade ≥3	Any Grade
	Number (Percent)					
Pulmonary embolism	0	0	2 (9.5%)	2 (9.5%)	0	0
Hypertension	3 (2.5%)	3 (2.5%)	0	6 (28.6%)	0	2 (5.3%)
Fatigue	22 (18.6%)	26 (22%)	1 (4.8%)	11 (52.4%)	0	1 (2.6%)
Dyspnea	11 (9.3%)	11 (9.3%)	0	1 (4.8%)	0	1 (2.6%)
Rash/Pruritus	10 (8.5%)	10 (8.5%)	0	1 (4.8%)	0	1 (2.6%)
Mucositis	5 (4.2%)	5 (4.2%)	1 (4.8%)	4 (19%)	0	0
Colitis	0	0	0	0	2 (5.3%)	2 (5.3%)
Cardiotoxicity	1 (0.8%)	1 (0.8%)	0	1 (4.8%)	1 (2.6%)	1 (2.6%)
Diarrhea	12 (10.2%)	15 (12.7%)	1 (4.8%)	4 (19%)	0	0
Anemia	9 (7.6%)	9 (7.6%)	0	0	0	5 (13.2%)
Hand-Feet-Syndrome	13 (11%)	14 (11.9%)	0	0	0	0
INR elevation	0	0	1 (4.8%)	1 (4.8%)	0	0
Nausea/Emesis	21 (17.8%)	24 (20.3%)	0	2 (9.5%)	2 (5.3%)	2 (5.3%)
irAE	0	0	0	0	2 (5.3%)	2 (5.3%)
Weight/appetite loss	6 (5.1%)	8 (6.8%)	0	7 (33.3%)	0	0
Proteinuria	0	0	0	2 (9.5%)	1 (2.6%)	1 (2.6%)
GIT-Bleeding	3 (2.5%)	3 (2.5%)	0	1 (4.8%)	1 (2.6%)	1 (2.6%)
Nosebleeds	1 (0.8%)	1 (0.8%)	0	0	0	0
Keratosis	1 (0.8%)	1 (0.8%)	0	0	0	0

**Notes**: We summarized urinary tract infections, pneumonias, fever and all other infections as infections; encephalopathia, dizziness, tremor, epileptic attacks and paresthesias as neurological AEs (Data are presented as absolute frequency with relative frequency in parenthesis.).

**Abbreviations**: Ire AE, Immune related adverse events; GIT-Bleeding, gastrointestinal bleeding.

## Discussion

In a real-world context, our study indicates that atezolizumab/bevacizumab (Atez/Bev) offers the best option for treating unresectable hepatocellular carcinoma independent of etiology, displaying the highest median Progression-Free Survival (PFS) and 15-month Overall Survival (OS), as well as the best objective response rate (ORR) particularly in patients with stable liver function or those who received prior Selective Internal Radiation Therapy (SIRT). Interestingly, lenvatinib proved to be non-inferior to Atez/Bev in terms of therapy response and PFS. Furthermore, sorafenib showed remarkable efficacy in patients with deteriorated liver function. Our data also underscored the predictive utility of the CRAFITY score for immunotherapy since it only worked for the Atez/Bev group.

This study emerges within a clinical landscape where the treatment of unresectable hepatocellular carcinoma has long been constrained to a single first-line treatment option, sorafenib.^2^ However, with the approval of further therapies, such as lenvatinib and atezolizumab/bevacizumab, the treatment landscape has highly expanded.^3^,^4^ Despite this progression, to our knowledge, real-world data comparing these three drugs in one cohort are missing. Moreover, the recent approval of tremelimumab/durvalumab offers a direction for further analysis of the comparative role of systemic therapies in patients with advanced HCC in a real-world setting.^5^ Furthermore, there are no randomized studies directly comparing lenvatinib vs immune checkpoint inhibitors and it is not expected that such a prospective trial will be performed. Therefore, our study aims to bridge this gap by comparing atezolizumab/bevacizumab to lenvatinib and to sorafenib and determining which patients might best benefit from each therapy, taking into account their individual condition and history, aiming for optimal patient outcomes.

Major prospective phase III clinical trials often excluded patients with deteriorated liver function and main vessel tumor infiltration, leading to uncertainty about the best treatment for these patients.^2–5^,^15^ Corresponding only 61.6% of our sorafenib cohort would have met the inclusion criteria for the SHARP-Study.^2^ Only 47.6% of our lenvatinib group would have met the inclusion criteria of the REFLECT-Study and 71.5% of the Atez/Bev group those of the IMBrave150 trial.^3^,^4^ Despite these exclusions, our real-world data shows comparable or even better outcomes in terms of tumor response.^16^ However, overall survival and progression-free survival were generally inferior to the phase III trials, likely due to the high proportion of patients in our study who would have been excluded from these trials.

Among the three groups of our cohort, the percentage of patients with Child-Pugh B/C status treated with atezolizumab/bevacizumab was similar to that with lenvatinib, while the sorafenib group had significantly more Child-Pugh B/C patients. There was no significant difference in the rate of patients with AFP ≥400ng/mL between the groups, and no significant difference in the reception of second-line therapy between the atezolizumab/bevacizumab and lenvatinib groups. In terms of efficacy among the three groups, atezolizumab/bevacizumab demonstrated the highest efficacy in our study, especially for tumor shrinkage. It outperformed lenvatinib significantly in terms of overall survival and was statistically superior to sorafenib in terms of OS, PFS and ORR, reflecting the findings of the IMBrave trial. Overall, our sorafenib group showed less efficacy than in the REFLECT and IMBrave studies, possibly attributed to the less favorable patient collective in terms of liver function but also to the lack of treatment alternatives prior to 2018. Lenvatinib, while mostly statistically non-inferior to atezolizumab/bevacizumab, showed the worst median overall survival and progression-free survival, likely due to the bad patient collective and its small cohort size. Only for OS Atez/Bev could also outperform lenvatinib significantly. These results are also underpinned by our univariate time to event analysis, which showed significant reduced hazard ratios for Atez/Bev compared to sorafenib but not for lenvatinib (Atez/Bev vs sorafenib: HR: 0.57, p = 0.03; vs lenvatinib: HR: 0.56, p = 0.10).

The fact that there has been no prospective randomized clinical trial comparing atezolizumab/bevacizumab (Atez/Bev) and lenvatinib to date underscores the relevance of our real-world findings. It is worth mentioning that a large meta-analysis comparing the most relevant phase III trials demonstrated the longest overall survival (OS) for the combination of immune checkpoint inhibitor with anti-VEGF (vascular endothelial growth factor), such as Atez/Bev.^17^ However, there was no statistically significant difference in objective response rate (ORR), OS, and progression-free survival (PFS) compared to lenvatinib, aligning with our study results. They also presented results of the LEAP-002 trial involving lenvatinib plus pembrolizumab showing the longest PFS of all phase III trials with another prolongation of OS around 2 months compared to the IMbrave-150 trial with slightly worse ORR.^18^ Although this study did not meet the predefined statistical significance, it reinforces the role of lenvatinib as a first-line treatment. These findings further highlight the efficacy of both Atez/Bev and lenvatinib for the treatment of hepatocellular carcinoma (HCC) in the first-line therapy and are in line with the outcomes in our real-world cohort of patients.

A distinctive feature of our study is the comparison of Atez/Bev to lenvatinib and sorafenib in one unselected cohort. Our findings gain credibility when compared to other real-world studies. For instance, the first meta-analysis of retrospective studies comparing Atez/Bev with lenvatinib found no statistically significant difference in treatment efficacy, mirroring our results, except for OS, which was significantly higher for Atez/Bev in our study.^19^ This study also noted that Atez/Bev performs less effectively in real-world situations than in phase III trials, a finding consistent with ours.

In contrast to our study, another recent German retrospective real-world study compared Atez/Bev (n = 100) to a combined group of sorafenib (n = 43) and lenvatinib (n = 37) without differentiating between the two, resulting in the statistically significant superiority of Atez/Bev over tyrosine kinase inhibitors (TKIs) in terms of ORR, OS and PFS.^20^ Despite a slightly higher number of BCLC C stage patients in their Atez/Bev group and fewer Child-Pugh class B and BCLC stage C patients in their TKI group, their patient collective was quite similar to ours in terms of baseline parameters, and the results were comparable. Hence, their findings reinforce ours, as Atez/Bev outperformed other groups in terms of ORR, OS, and PFS, and OS, just like PFS, decreased with worsening liver function.

In our subgroup analysis, we compared the progression-free survival (PFS) of patients, accounting for liver function, etiology, or previous local treatment. All therapies exhibited longer PFS with improved liver function at the beginning of the treatment, but in comparison, sorafenib was inferior to the other drugs. However, sorafenib was not inferior for patients with decreased liver function when compared to the other groups, showing even the longest PFS in these patients. In this context, we also wish to highlight the results of the univariate time-to-event analysis for PFS in patients treated with sorafenib. Although the median hazard ratio (HR) was <1 for higher Child-Pugh and ALBI scores, these results were not statistically significant. This observation aligns with the results of large cohort studies, such as the GIDEON study, which demonstrated that sorafenib is a valid treatment option for patients with impaired liver function.^21^,^22^

The selection of patients for immunotherapy, particularly based on etiology, is controversially discussed to date. The above mentioned meta-analysis comparing Atez/Bev with lenvatinib stated that Atez/Bev is more favorable for HCC caused by viral hepatitis, whereas lenvatinib is more favorable for patients with Child-Pugh class B and non-viral etiologies, such as NASH.^19^ In contrast, we observed no relevant influence on the PFS in patients treated with Atez/Bev across different etiologies, but the shortest PFS was observed in patients with hepatitis B, aligning with the latest published posthoc analysis of IMbrave150 study.^23^ Nevertheless, the study shows a disease control rate of 72.8% for patients with hepatitis B. In our cohort of patients, we observed high efficacy of Atez/Bev especially for non-viral HCC such as NASH, as we recorded the longest PFS compared to the other treatments. Thus, the benefit of Atez/Bev for HCC with NASH etiology remains contentious. Some studies suggest a diminished efficacy of immunotherapy in this patient group, potentially due to deviant T-cell activation associated with NASH, leading to lower responsiveness and consequently, no survival benefit for Atez/Bev in NASH patients.^19^,^24–26^ Interestingly, these findings have not been corroborated in recent phase III trials, such as the HIMALAYA and LEAP-002 studies, nor in other retrospective real-world studies we previously mentioned.^4^,^18^,^20^

Given that the meta-analysis did not evaluate prior local therapies at all and the other German study merely recorded but did not differentiate them, we aimed to analyze deeper on the influence of prior local therapies on the outcome of first-line therapies. We found that previous therapies could indeed impact therapy outcomes. Particularly, Selective Internal Radiation Therapy (SIRT) exhibited a statistically significant longer PFS with Atezolizumab/Bevacizumab (Atez/Bev), probably due to its enhancement of activated CD8+ T-cell presence within the tumor microenvironment, which augments the effects of Atez/Bev.^27^ However, as we only had one patient in the lenvatinib group who received SIRT, a reliable comparison is not feasible. The NASIR-HCC Phase II trial also showed convincing results regarding the combination of SIRT with immunotherapy.^28^ Similarly, the abstract publication of the ongoing IMMUTACE phase II trial shows a favorable impact on the Overall Response Rate (ORR) with Transarterial Chemoembolization (TACE) prior to immunotherapy.^29^ In our study, we observed the longest PFS in patients from the TACE subgroup who were treated with Atez/Bev, although this difference was not statistically significant. The effects of local therapies could provide intriguing directions for further evaluations in the context of individual therapy decisions. Several studies to evaluate sequential or additive immune checkpoint inhibitors with SIRT or TACE are already ongoing and will reveal the role of local therapy for the therapy outcome in the next years.

Our univariate time-to-event analysis revealed a hazard ratio (HR) of 2.65 (p = 0.01) for a higher CRAFITY-Score among the patients. Interestingly, this was the only statistically significant parameter we could identify that predicts progression-free survival (PFS). The CRAFITY-Score, developed and published in October 2021, is associated with survival and response rates in patients receiving PD-L1 immunotherapy.^30^ Our findings lend further real-world support to this score.

In line with other studies, we found that the tolerability of Atez/Bev was significantly better compared to the other groups, which makes it an even more compelling treatment option.^19^,^20^ We also want to clarify that symptoms of liver failure, such as ascites, total bilirubin elevation, etc., were not classified as adverse events. This is because it was not clearly definable which symptoms were treatment-related or due to disease progression, particularly in the sorafenib group with 39.8% of patients being classified as Child-Pugh B. For better comparability, this should be considered in future assessments.

We would like to emphasize the strengths of this study, as to the best of our knowledge, it represents the first study comparing three first-line therapies with large cohorts for sorafenib (118 patients) and Atez/Bev (38 patients). Due to this fact our results are of particular interest as we gain information about the outcome of all three therapies in the same cohort of patients in a real-world setting and this information can be helpful in the making of therapy decision.

Limitations of our study are the retrospective design lacking randomization, the heterogeneity of the compared groups, particularly given the small size of our lenvatinib cohort (21 patients) and the monocentric study design. Moreover, before 2018, sorafenib was the only therapy option and no second-line treatments were available, which may have potentially skewed outcomes of patients treated with sorafenib. Also the statistically significant better BCLC of Atez/Bev compared to the other groups, just like the compared to sorafenib better CHILD-PUGH-Class might have an impact on the results, although the univariate analysis showed no significancy for these parameters. Another limitation is the lack of data on quality of life. We cannot exclude that all these limitations have an impact on the results.

In conclusion, the treatment of unresectable hepatocellular carcinoma with atezolizumab plus bevacizumab not only demonstrates the longest progression-free and overall survival, particularly in patients with stable liver function, but it also has lower rates of severe adverse events in our cohort of unselected patients. Based on our retrospective study, Atez/Bev confirms to be the best therapy of choice for patients with good liver function and a CRAFITY Score of 0, regardless of etiology. Lenvatinib appears to be a viable alternative for patients unsuitable for Atez/Bev treatment. For patients with impaired liver function, sorafenib remains a possible treatment as it was non-inferior to the other groups, albeit with a higher frequency of severe adverse events. Therefore, other therapies should be preferred when feasible. The observed effects warrant further prospective investigations. Given the inherent limitations of a retrospective study in comparison to other study designs, more evidence-based therapy decisions for the individual and the potential of further immunotherapies, such as tremelimumab and durvalumab, are required for the evolving landscape of HCC treatment.^31^

## Abbreviations

AE, adverse events; Atez/Bev, atezolizumab plus bevacizumab; BCLC, Barcelona Clinic Liver Cancer; DCR, disease control rate; ECOG, Eastern Cooperative Oncology Group; GIT-Bleeding, gastrointestinal bleeding; HIFU, high-intensity focused ultrasound; IrAE, immune related adverse events; MWA, microwave ablation; ORR, objective response rate; OS, overall survival; PFS, progression free survival; RFA, Radiofrequency Ablation; SIRT, selective internal radiation therapy; TACE, transarterial chemoembolization; TIPS, transjugular intrahepatic portosystemic shunt; TKI, tyrosine-kinase-inhibitor.

## Lay Summary

This study compares three first-line treatments for advanced liver cancer, revealing atezolizumab/bevacizumab as most effective, especially in patients with stable liver function or prior local treatment. Lenvatinib achieved similar objective response, while sorafenib seemed to work best for patients with deteriorated liver function.
